# Racial differences in quantitative background parenchymal enhancement on breast magnetic resonance imaging

**DOI:** 10.1002/cncr.70174

**Published:** 2025-11-16

**Authors:** Mattia A. Mahmoud, Christine E. Edmonds, Bruno Barufaldi, Alex Nguyen, Oluwadamilola M. Fayanju, Routiakou Sore, Sarah Ehsan, Carla R. Zeballos Torrez, Jinbo Chen, Despina Kontos, Anne Marie McCarthy

**Affiliations:** ^1^ Department of Biostatistics, Epidemiology, & Informatics Perelman School of Medicine University of Pennsylvania Philadelphia Pennsylvania USA; ^2^ Abramson Cancer Center Penn Medicine Philadelphia Pennsylvania USA; ^3^ Department of Radiology Perelman School of Medicine University of Pennsylvania Philadelphia Pennsylvania USA; ^4^ Department of Surgery Perelman School of Medicine University of Pennsylvania Philadelphia Pennsylvania USA; ^5^ Penn Center for Cancer Care Innovation Abramson Cancer Center Penn Medicine Philadelphia Pennsylvania USA; ^6^ The Leonard Davis Institute of Health Economics University of Pennsylvania Philadelphia Pennsylvania USA; ^7^ Department of Radiology Columbia University Irving Medical Center New York New York USA

**Keywords:** BI‐RADS, breast density, MRI, quantitative BPE, race

## Abstract

**Importance:**

Although Black women have higher absolute quantitative breast density, they are often assigned lower subjectively determined Breast Imaging and Reporting Data System (BI‐RADS) density scores than White women. Background parenchymal enhancement (BPE) on breast magnetic resonance imaging is independently linked to breast cancer risk and may improve risk stratification for Black and White women.

**Objective:**

To evaluate differences in quantitative BPE between Black and White women and determine whether breast cancer risk factors mediate these differences.

**Design, Setting, and Participants:**

A cross‐sectional study of 1202 women (200 Black, 1002 White; aged 40–74 years) with negative mammograms and no breast cancer history who underwent breast magnetic resonance imaging between 2016 and 2023 at an academic medical center.

**Exposures:**

Self‐reported race (Black vs. White).

**Main Outcomes and Measures:**

The primary outcome was automated, quantitative BPE (median BPE and BPE ratio). Covariates included BI‐RADS density, fibroglandular tissue volume, qualitative BPE, age, body mass index, and menopausal status.

**Results:**

Fewer Black women were classified as having extremely dense breasts (10% vs. 21%; *p* < .01), yet similar proportions had high qualitative BPE (35% vs. 29%; *p* = .29). Quantitative BPE was significantly higher in Black women (median difference, 1.51; standard deviation, 9; 95% CI, 0.13–2.90), independent of covariates. No risk factors mediated this difference.

**Conclusions and Relevance:**

Despite lower BI‐RADS density in Black women, as suggested by prior literature, higher quantitative BPE was found, suggesting that BPE captures aspects of breast tissue composition not reflected by density. Future studies can incorporate BPE into risk models, which can improve performance and reduce disparities in risk prediction.

## Abstract 2

Black women had higher median quantitative background parenchymal enhancement levels compared to White women, when adjusted for age, menopausal status, body mass index, and fibroglandular tissue volume. None of those covariates explained a significant proportion of the racial differences in quantitative background parenchymal enhancement.

## INTRODUCTION

Background parenchymal enhancement (BPE) is an imaging biomarker for breast cancer that has been shown to have a stronger association with breast cancer risk than breast density.^1^, ^2^ During a dynamic contrast‐enhanced magnetic resonance imaging (MRI) of the breasts, images are obtained before and after intravenous injection of a gadolinium‐based contrast agent. This technique enables visualization of lesions with vascularity, including the vast majority of breast malignancies. In addition to the enhancement of breast masses and other focal lesions, normal fibroglandular tissue (FGT) can also enhance, a phenomenon known as BPE.^3^ Although the precise pathophysiology of BPE is not entirely known, research suggests that BPE distinguishes nonhormonally active fibrous breast tissue from hormonally active glandular tissue, with levels of enhancement greatly varying between individuals.^3^ Moreover, higher levels of BPE have been associated with obesity and elevated endogenous hormone levels, particularly in women at elevated risk for breast cancer.^4^

Clinical assessment of BPE involves assignment of a qualitative category assigned by the interpreting radiologist according to the American College of Radiology Breast Imaging and Reporting Data System (BI‐RADS) classification system, with four ordinal levels of increasing enhancement: minimal, mild, moderate, and marked.^5^ Despite being the standard of care, qualitative BPE assessments can be subjective because they rely on visual interpretation by radiologists and are associated with high inter‐ and intra‐reader variability.^5^ To address this, prior studies have calculated quantitative BPE levels using automated image segmentation.^6^ Using a fully automated method, one study of 50 *BRCA* carriers demonstrated that quantitative BPE levels significantly decreased after risk‐reducing salpingo‐oophorectomy, with these imaging changes offering a potential indicator of the reduced breast cancer risk conferred by risk‐reducing salpingo‐oophorectomy.^7^ More recently, fully automated quantitative BPE showed a greater association between high BPE and breast cancer risk than qualitative BI‐RADS assessments in the same population of women.^8^, ^9^ Thus quantitative BPE measures can provide a more accurate and reliable measurement of BPE, a measure that may improve breast cancer risk assessment and ultimately guide high‐risk screening and risk‐reduction strategies.

Structural racism, not race, drives racial health disparities. Nevertheless, just as rates of obesity of differ between racial groups, other breast cancer risk factors such as breast density have also been shown to differ by race.^10^ While subjective BI‐RADS assessments generally show that Black women have lower percent breast density than White women—likely because of the association between body mass index (BMI) and breast density—quantitative measures, such as dense area or volume, help capture that Black women have similar or even higher absolute quantity of dense tissue.^11^, ^12^ Because clinical practice and risk assessment tools rely heavily on BI‐RADS density, which may disadvantage Black women, it is critical to explore additional biomarkers like BPE. This is especially important given that Black women are more likely to be diagnosed at a younger age, present with more aggressive disease, and experience higher mortality rates.^13^, ^14^, ^15^ To summarize, although race is a poor surrogate for biological differences, it remains a marker of persistent inequities in breast cancer screening, treatment, and outcomes.^13^

Thus, the purpose of this study was to compare quantitative BPE levels by race, and if differences do exist, use a causal mediation analysis approach to evaluate if those differences are mediated by breast cancer risk factors such as age, menopausal status, FGT, and BMI.^2^, ^4^, ^16^ We hypothesize that, similar to quantitative breast density, Black women will have higher quantitative BPE levels. This serves as a preliminary step toward investigating BPE as a potential biomarker of breast cancer risk among Black women.

## MATERIALS AND METHODS

Self‐identified Black and White women who had both a mammogram and a subsequent MRI at one of four sites within our health system between 2016 and 2023 were included in this study. Only mammograms with an initial BI‐RADS assessment score of 1 (negative) or 2 (benign finding) were retained to focus on a population with no abnormalities in enhancement that may confound BPE assessment.

**FIGURE 1:**
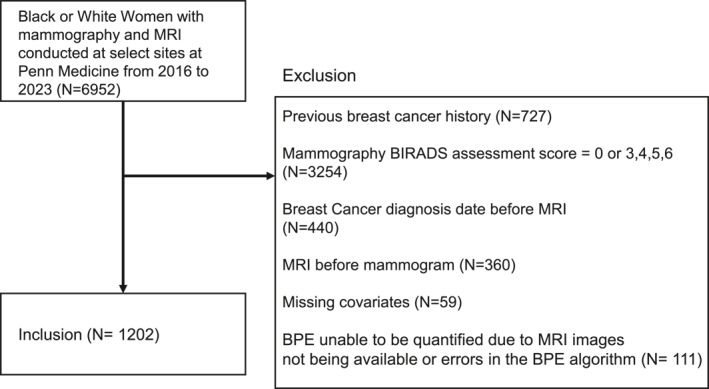
Inclusion/exclusion criteria.

Patients were excluded if they had a history of breast cancer before the included MRI; an initial BI‐RADS assessment score of 0 (abnormal/incomplete), 3 (probably benign), 4 (suspicious for malignancy), 5 (highly suspicious for malignancy), and 6 (known diagnosis of malignancy); missing covariates or quantitative BPE measures not available because of absence of necessary MRI sequences of images; or errors in the BPE algorithm (Figure 1).

The study was Health Insurance Portability and Accountability Act–compliant and approved by the institutional review board of the University of Pennsylvania with blinding on initial submission. A waiver of informed consent was granted for this review of existing clinical data.

### Image analysis

Information on the MRI examination protocol can be found in the supplement. We used a publicly available 3D U‐Net pipeline to segment the breast and FGT from T1‐weighted fat‐suppressed precontrast MRI.^17^ The method uses two sequential 3D U‐Nets: one for breast and one for FGT segmentation. Pretrained weights and code were used as provided.^17^, ^18^, ^19^, ^20^, ^21^ The segmented breast mask was used to register the first postcontrast MRI sequence with the precontrast scan.^22^ The segmented FGT mask was used to estimate quantitative BPE. This BPE measurement is estimated based on tissue enhancement after contrast agent introduction (i.e., first postcontrast sequence image) versus before (precontrast) using an enhancement ratio (ER) defined as

$$ER=\frac{\left(i_{POST}-i_{PRE}\right)}{i_{PRE}+\varepsilon }$$

where *i*_*PRE*_ is precontrast enhancement and *i*_*POST*_ is post‐BPE voxels (three‐dimensional equivalent of pixels) are then defined as those within the FGT with ER above an arbitrary cutoff.^18^, ^23^, ^24^ The cutoff of 1.2 for enhancing/nonenhancing was chosen as that which best correlated with qualitative BPE in previous work.^8^ The factor ε prevents division by zero in the formulation. We chose median BPE and the BPE ratio volume as our quantitative BPE measures of interest. Median BPE is unitless and is the median enhancement ratio over all voxels in the FGT, a ratio in [0, ∞), and is less sensitive to outliers compared to mean BPE. BPE/FGT is the proportion of voxels with ER ≥ 1.20, of voxels within the FGT volume, a proportion in [0, 1].

### Statistical analysis

At the time of mammography, we ascertained covariates including age, race/ethnicity, menopausal status, BMI, and BI‐RADS density. We assessed BI‐RADS–based qualitative BPE on MRI. We extracted FGT using the MRI segmentation algorithm detailed previously. We assessed the distributions of BPE and covariates by race and tested for differences using chi‐square and *t*‐tests, as appropriate. Additionally, we performed linear regression analyses with race/ethnicity as the primary exposure and median BPE or BPE ratio as the primary outcome, adjusting for age, BMI, menopausal status, and FGT. We report both the unstandardized and standardized coefficients in standard deviation units.

We conducted a causal mediation analysis to explore the extent to which specific breast cancer risk factors (age, BMI, FGT, and menopausal status) separately explain potential racial differences in quantitative BPE. In this mediation analysis, we defined race as the exposure and treated each quantitative BPE measure as the outcome in separate models. We included age, BMI, FGT, and menopausal status as confounders when not tested as mediators.

We focused on the counterfactual disparity measure (CDM) as the primary measure of interest in the mediation analysis. The CDM, which is similar to the controlled direct effect, compares the risk of high BPE levels between Black women and White women while holding a mediator fixed at a set value.^25^, ^26^ The CDM quantifies the magnitude of the association between race/ethnicity and BPE that would remain if we fixed a specific mediator uniformly for both Black and White women in the population. For example, we interpreted the CDM as the racial difference in BPE levels that would persist even after intervening on BMI. Of note, we rely on self‐identified racial categories to capture the historical and ongoing socioeconomic and political forces that shape the experiences of women in our cohort, rather than to imply any inherent biological differences.^13^

We tested age, BMI, FGT, and menopausal status as potential mediators. To assess the CDM, we assumed no uncontrolled exposure‐outcome confounding and no mediator‐outcome confounding. We used a minimally sufficient set of variables to control for potential confounding in both the exposure‐outcome and mediator‐outcome relationships. We conducted a complete case analysis to handle missing covariates. We performed all statistical analyses using R version 4.1.2.^27^ We used a two‐sided alpha of 0.05 to assess statistical significance.

## RESULTS

We included 200 Black and 1002 White women in the analytic study population, with one MRI per patient included. Overall, Black women were younger at the time of mammogram (47.4 vs 49.0, *p* = .03), were more likely to be premenopausal (74% vs 65%, *p* = .01), and less likely to have heterogeneously dense or extremely dense levels (57% vs 77%, *p* < .01) [Table 1]. Additionally, Black women had greater total breast volume (3060 cm^3^ vs 2034 cm^3^, *p* < .01), median BPE levels (9.43 vs 7.76, *p* = .03), and BPE ratio percentages (25% vs 22%, *p* = .01). Black and White women had similar BMI, radiologist‐rated BPE levels, and FGT. Finally, Black women had a lower percentage of women undergoing screening MRIs (72% vs 89%, *p* < .01). There was no statistically significant association between screening status and either quantitative BPE measure. According to the Student *t*‐test, mean differences in screening (0.222) vs diagnostic (0.227) for BPE ratio had a *p* value of .70, whereas mean differences in screening (8.24) vs diagnostic (7.85) for median BPE had a *p* value of .61.

**TABLE 1:** Demographic characteristics by race.

Characteristics	Black women (*N* = 200)	White women (*N* = 1002)	*p*
Age at mammogram (mean, SD)	47.4 (9.56)	49.0 (11.3)	.03
Body mass index (mean, SD)	29.8 (6.8)	30.9 (8.2)	.89
Menopausal status (*N*, %)			.01
Premenopausal	148 (74%)	646 (65%)	
Postmenopausal	52 (26%)	356 (35%)	
Breast Imaging Reporting and Data System breast density (*N*, %)			<.01
Entirely fatty ‐ A	12 (6%)	13 (1%)	
Scattered fibroglandular tissue ‐ B	73 (37%)	221 (22%)	
Heterogeneously dense ‐ C	94 (47%)	567 (57%)	
Extremely dense ‐ D	21 (10%)	201 (20%)	
Qualitative background parenchymal enhancement (BPE) (*N*, %)			.29
Minimal	34 (17%)	222 (22%)	
Mild	97 (49%)	486 (49%)	
Moderate	50 (25%)	220 (22%)	
Marked	19 (10%)	74 (7%)	
Cancer status			.06
No	190 (95%)	979 (98%)	
Yes	10 (5%)	23 (2%)	
Breast volume (cm^3^, mean [SD])	3060 (1632)	2034 (1125)	<.01
Fibroglandular tissue (cm^3^, mean [SD])	202 (136)	194 (133)	.43
Median BPE (mean, [SD])	9.43 (9.92)	7.76 (9.03)	.03
BPE ratio (%, mean, [SD])	25 (19)	22 (17)	.01
Magnetic resonance imaging status			<.01
Screening	144 (72%)	113 (89%)	
Diagnostic	56 (28%)	889 (11%)	

**FIGURE 2:**
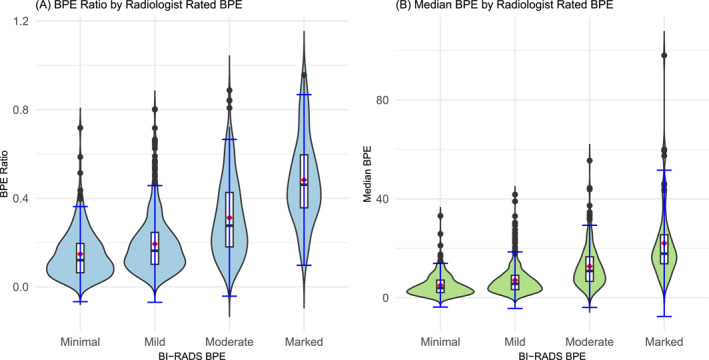
Violin plots show distribution of (A) background parenchymal enhancement (BPE) ratio and (B) median BPE within categories of subjective Breast Imaging Reporting and Data System (BI‐RADS), radiologist‐rated BPE.

Higher qualitative BPE levels were associated with higher quantitative BPE levels, whereas mammographic BI‐RADS breast density and quantitative BPE levels were not associated, even when stratified by race (Figure 2, Supplemental Table 1). Specifically, women with marked BPE had the highest median BPE (19.7 [SD = 16.2]) and BPE ratio (46.0 [SD = 21.2]), whereas women with minimal BPE had the lowest median BPE (4.06 [SD = 5.63]) and BPE ratio (0.14 [SD = 0.11]), *p* < .01. There were no significant differences in median BPE and BPE ratio between BI‐RADS breast density categories, regardless of stratification by race.

Black women were found to have significantly higher quantitative BPE even after adjusting for BMI, age, menopausal status, and FGT (Table 2). Age and menopausal status were inversely associated with BPE, an association that was statistically significant (*p* < .01). Higher FGT was associated with higher median BPE (*p* < .01), whereas BMI was not significantly associated with median BPE. Finally, the covariates in their respective models explained 10% of the variance in median BPE and 11% of the variance in the BPE ratio.

**TABLE 2:** Linear regression models of race/ethnicity and quantitative BPE.

	Median BPE		BPE ratio	
	Unstandardized Β_1_, (95% CI) [*p* value]	Standardized Β_1_, (95% CI) [*p* value]	Unstandardized Β_1_, (95% CI) [*p* value]	Standardized Β_1_, (95% CI) [*p* value]
Adjusted				
Race/ethnicity				
Black women	1.95 (0.61–3.29) [<.01]^a^	—	0.04 (9.63e‐3 to 0.06) [<.01]^a^	—
White women	REFERENCE	—	REFERENCE	—
Race/ethnicity				
Black women	1.51 (0.13–2.90) [.03]^a^	0.18 (0.02–0.33) [.026]	0.03 (–2.8e‐4 to 0.05) [.05]	—
White women	REFERENCE	REFERENCE	REFERENCE	—
BMI (per 1 kg/m^2^)	–2.27e‐4 (–4.13e‐3 to 3.67e‐3) [.90]	–3.41e‐3 (–0.06 to 0.06) [0.90]	3.63e‐5 (–4.03e‐5 to 1.13e‐4) [.35]	0.03 (–0.03 to 0.08) [.35]
Age (per 1 year)	–0.11 (–0.19 to –0.03) [<.01]^a^	–0.14 (–0.25 to –0.04) [<.01]^a^	–2.47e‐3 (–4.02e‐3 to –9.21e‐4) [<.01]^a^	–0.16 (–0.26 to –0.06) [<.01]^a^
Menopausal status				
Premenopausal	REFERENCE	REFERENCE	REFERENCE	—
Postmenopausal	–2.63 (–4.56 to –0.70) [<.01]^a^	–0.28 (–0.49 to –0.06) [.012]	–5.51e‐2 (9.14e‐2 to –0.02) [<.01]^a^	—
FGT volume (per 1 cm^3^)	6.04e‐3 (2.23e‐3–9.88e‐3) [<.01]^a^	0.10 (0.04–0.16) [<.01]^a^	4.44e‐5 (‐2.95e‐5–1.18e‐4) [.24]	0.04 (–0.02 to 0.09) [.24]

*Note:* Adjusted for age, BMI, menopausal status, and FGT volume.

Abbreviations: BMI, body mass index; BPE, background parenchymal enhancement; FGT, fibroglandular tissue.

^a^ Denotes statistical significance.

To account for the right‐skewed distribution of BPE measures (Supplemental Figure 1 and Figure 2), we conducted additional linear regression analyses using log‐transformed median BPE and BPE ratio (Supplemental Figure 3 and Figure 4) as outcomes (Supplemental Table 2). Although age remained consistently and significantly inversely associated with both log‐transformed outcomes (*p* < .001), other associations shifted. For example, Black women continued to show significantly higher log‐transformed median BPE (*β* = 0.22; 95% CI, 0.07–0.38; *p* = .003), but the association with log‐transformed BPE ratio was no longer statistically significant (*β* = 0.077; 95% CI, –0.052 to 0.206; *p* = .24). Postmenopausal status was significantly associated with lower log‐transformed BPE ratio (*β* = –0.20; 95% CI, –0.38 to –0.02; *p* = .03), but its association with log‐transformed median BPE was weaker and not statistically significant (*p* = .10). FGT volume, which was significantly associated with median BPE on the original scale, was not significant in the log‐transformed model (*p* = .19).

When conducting the causal mediation analysis, eliminating differences in BMI, age, FGT, or menopausal status at the time of breast imaging between Black and White women did not result in a significantly different estimated counterfactual disparity in BPE ratio or median BPE levels on the risk difference scale [CDM_ratio_ = 0.03 (95% CI, –2.53e‐4 to 5.21e‐2; *p* = .05), CDM_median_ = 1.16 (95% CI, –0.24 to 2.57; *p* = .10)]. Less than 0.1% of the racial differences in either BPE measure could be explained by BMI or age. FGT explained 1% of the racial differences in median BPE and 0.5% in BPE ratio. Menopausal status explained 9% of the racial differences in median BPE and 8% in BPE ratio.

## DISCUSSION

To our knowledge, this is the first investigation to examine and compare quantitative BPE levels among Black and White women. Qualitative BPE was slightly higher among Black compared to White women, but not statistically different. However, Black women had statistically significantly higher levels of quantitative BPE measures than White women, both unadjusted and when adjusted for age, menopausal status, BMI, and FGT.

This novel study examined quantitative BPE levels among Black women to explore potential Black–White racial differences in quantitative BPE within a diverse study population. Fewer Black women had extremely dense breasts compared to White women (10% vs 20%, *p* ≤ .01), and there was not a correlation between BI‐RADS breast density and quantitative BPE levels regardless of race. As expected, there was a positive correlation between the qualitative and quantitative BPE measures in our study population. Importantly, even if the rates of obesity were comparable between Black and White women—which is not the case, given that the overall prevalence of obesity is higher among Black women—Black women would still have higher quantitative BPE levels to White women, after adjusting for age, BMI, menopausal status, and FGT.

Although no other studies have reported racial differences in quantitative BPE, previous studies have examined the association between various BPE measures, age, menopausal status, and density. Watt et al. (2023) also found that both the median BPE and BPE_20_/FGT values had positive correlation with qualitative BPE levels.^8^ Additionally, they showed that age and menopausal status had a statistically significant inverse relationship with quantitative BPE.^8^ Although they also found that density, specifically BI‐RADS FGT, was uncorrelated with BPE, other studies have shown mixed results on the BPE–breast density relationship.^28^, ^29^

Although we observed racial differences in quantitative BPE levels, it is noteworthy that age, BMI, menopausal status, and FGT individually did not significantly mediate any racial variation in BPE levels. This is surprising given the extensive prior literature detailing Black–White differences in obesity, age at menopause onset, and both qualitative and quantitative breast density.^10^, ^16^, ^30^ There are relatively few studies examining racial differences in quantitative breast density measures; however, the majority of research to date suggests that Black women have lower relative breast density (as measured by volumetric or area percent density) but higher absolute breast density (as measured by dense area or volume) compared to White women.^31^, ^32^, ^33^ This may reflect that BPE captures additional biologic processes—such as vascularity—not fully explained by these variables. Unmeasured factors, including structural and social determinants of health, reproductive factors such as parity or age at menarche, or environmental exposures, may contribute more strongly to racial differences in BPE than the mediators assessed in this study. Furthermore, despite having a large population of Black women undergoing mammography screening, few Black women in our institutionally sourced cohort had MRIs, a trend we have also observed and previously described among women at our institution who have actually had breast cancer.^34^

Although the absolute differences in median BPE between Black and White women (e.g., 9.4 vs. 7.8 for median BPE; 25% vs. 22% for BPE ratio) may appear modest, they are consistent with prior reports of racial differences in quantitative breast density measures (Black women have higher absolute breast density), which have varying differences in magnitude between studies but appear as clinically meaningful at the population level. Importantly, we emphasize that the magnitude of these differences, rather than statistical significance alone, is most relevant for interpretation. Thus, we interpret our findings as evidence that racial differences in BPE exist but may be limited in size, in contrast to the more pronounced disparities observed for some other breast cancer risk factors.

BPE may be a strong independent risk factor for breast cancer in Black women and should be considered as a biomarker to improve risk stratification. However, the discussion surrounding BPE should always be contextualized within the framework of preexisting racial disparities in the utilization of screening MRI. Black women are less likely to be eligible, and even if eligible, less likely to receive supplemental or high‐risk breast MRI screening.^10^, ^17^, ^18^, ^19^, ^20^, ^23^, ^24^, ^25^, ^26^, ^27^, ^28^, ^29^, ^30^, ^35^, ^36^, ^37^ The question of whether BPE is a valuable biomarker will have evolving implications as a result of many U.S. states passing legislation mandating insurance coverage of supplemental screening, including breast MRI, for women with dense breasts.^38^, ^39^ Racial differences in eligibility and use of supplemental screening may change with expanded insurance coverage.^40^ However, given the lower prevalence of dense breasts among Black women, we expect these racial differences in eligibility and use based on breast density to persist.

There are several limitations to this study. First, we were only able to calculate BPE metrics for patients that had both a T1‐weighted non–fat‐saturated MRI scan and dynamic contrast‐enhanced–MRI scan. Some patients were excluded because of having imaging processing failures due to having implants, artifacts on MRI, large masses, or having failed processing. Even using a fully automated method to quantify precise BPE measurements, there could be true differences in BPE by race between differing study populations, as shown by the wide distribution of quantitative BPE levels between other studies using the same fully automated quantitative method.^8^, ^36^ This observation could be due to differences in quality control protocols after image segmentation and quantification between research groups or even the racial composition of the groups. Future work should continue to improve the accuracy and reproducibility of segmentation methods. Second, we used a subset of MRI scans originally collected as part of a separate study, which were limited to cases with initial BI‐RADS assessment scores of 1 or 2. As a result, our sample may not fully represent the broader population of women undergoing breast MRI, potentially introducing selection bias.

Finally, while we performed causal mediation analyses with respect to race as an “exposure,” we do not view race as an innate, biological characteristic of an individual.^41^ Although our study was not able to include covariates such as educational attainment, insurance status, income, or other measures that could serve as surrogates for social marginalization, it is important that future research consider exposures that more directly reflect membership in socially marginalized versus privileged groups, such as measures of structural racism, rather than relying solely on self‐identified racial identity.^41^

## CALL TO ACTION

Our findings underscore the critical need to examine BPE as a potential biomarker of breast cancer risk, particularly among Black women. First, research is warranted to explore the association between BPE and breast cancer risk across different racial groups. Second, a more nuanced understanding of the individual and combined roles of BPE, obesity, menopausal status, and breast density in breast tumorigenesis could help clarify how BPE and breast density independently contribute to breast cancer risk. Currently, our ability to accurately distinguish between individuals at high, intermediate, and average risk of breast cancer remains limited, as does our ability to assess risk over specified timeframes (ie, 5‐year risk as opposed to overall lifetime risk). Alarmingly, Black women continue to experience higher mortality rates from invasive breast cancer compared to White women and are diagnosed at younger ages.^2^ These findings highlight the urgent need to investigate additional biomarkers—such as BPE—to help address and ultimately reduce Black–White racial disparities in breast cancer outcomes.

## CONCLUSIONS

Black women exhibited higher quantitative BPE levels compared to White women. Despite the well‐documented relationships between race, age at menopause onset, and the prevalence of obesity, BMI, menopausal status, age, and FGT did not mediate a significant portion of the observed racial differences in quantitative BPE in our study. Our findings provide novel evidence that Black women have higher BPE levels than White women, despite having lower BI‐RADS breast density. Given the racial disparities in breast cancer detection, risk, and mortality, it is essential to explore new biomarkers such as BPE for their potential to better prognosticate breast cancer risk among Black women.

## AUTHOR CONTRIBUTIONS

**Mattia A. Mahmoud**: Conceptualization; Formal analysis; Funding acquisition; Investigation; Methodology; Project administration; Software; Validation; Visualization; Writing ‐ original draft; and Writing ‐ review & editing. **Christine E. Edmonds**: Conceptualization; Methodology; Visualization; Writing ‐ original draft; and Writing ‐ review & editing. **Bruno Barufaldi**: Formal analysis; Data curation; Investigation; Resources; Software; Validation; Visualization; and Writing ‐ review & editing. **Alex Nguyen**: Data curation; Formal analysis; Investigation; Resources; Software; Validation; and Writing ‐ review & editing. **Oluwadamilola M. Fayanju**: Conceptualization; Methodology; Writing ‐ original draft; and Writing ‐ review & editing. **Routiakou Sore**: Data curation; Investigation; Project administration; and Writing ‐ review & editing. **Sarah Ehsan**: Data curation; Investigation; Project administration; and Writing ‐ review & editing. **Carla R. Zeballos Torrez**: Visualization; Writing ‐ original draft; and Writing ‐ review & editing. **Jinbo Chen**: Conceptualization; Methodology; and Writing ‐ review & editing. **Despina Kontos**: Data curation; Investigation; Resources; Software; Writing ‐ review & editing; and Supervision. **Anne Marie McCarthy**: Conceptualization; Funding acquisition; Investigation; Methodology; Project administration; Resources; Supervision; Writing ‐ original draft; and Writing ‐ review & editing.

## CONFLICT OF INTEREST STATEMENT

The authors declare no conflicts of interest.

## Supporting information

Supplementary Material

## Data Availability

Data generated or analyzed during the study are available from the corresponding author by request.
