# Supplementary material for: Racial differences in quantitative background parenchymal enhancement on breast magnetic resonance imaging
Source: Cancer. 2025 Nov 16;131(22):e70174. doi: 10.1002/cncr.70174 (PMC12619913; doi:10.1002/cncr.70174)

SUPPLEMENTAL DOCUMENT

MRI examinations

Both full protocol MRIs and abbreviated MRIs (AB-MRI) were included in this study. All BI-RADS breast density and radiologist rated BPE values were obtained from studies that were interpreted by breast radiology fellowship-trained physicians or equivalent, using the terminology of the BI-RADS atlas. Full protocol MRIs included the following sequences: localizer sequence, axial T1 nonfat-saturated, axial T2-weighted short tau inversion recovery (STIR), axial T1 fat-saturated, three axial dynamic postcontrast T1 fat-saturated (imaging at injection, 90 seconds after injection, and 6.5 minutes after injection), and diffusion-weighted imaging. Subtraction sequences were created from the pre- and post-contrast images. Sagittal reformats and maximum intensity projections were also created from the subtraction sets. Abbreviated MRIs included the following sequences in the axial plane: axial STIR, T1 fat-saturated, one post-contrast T1 fat-saturated imaging. Subtracted and sagittal images were created from the post-contrast imaging. MultiHance (gadobenate dimeglumine) (Bracco Diagnostics, Princeton, NJ) was injected at 2 mL·s-1 (0.1 mmol·kg-1) followed by a 20-mL saline flush for all studies. All studies were interpreted on the same type of workstation (sectra, SECTRA, Linkoping, Sweden).

Supplemental Table 1. Comparison of BPE and Breast Density Levels Between Black and White Women

|  | Quantitative Background Parenchymal Enhancement | | | | | | | |
| --- | --- | --- | --- | --- | --- | --- | --- | --- |
|  | Black Women | | | | White Women | | | |
|  | Median BPE, mean (SD) | P value | BPE ratio, mean (SD) | P value | Median BPE, mean (SD) | P value | BPE ratio, mean (SD) | P value |
| Qualitative BPE |  | <0.01 |  | <0.01 |  | <0.01 |  | <0.01 |
| Minimal | 4.76 (3.95) | -- | 13.3 (8.93) | -- | 3.96 (5.85) | -- | 14.1 (10.8) | -- |
| Mild | 7.02 (8.92) | -- | 20.6 (15.4) | -- | 5.77 (6.00) | -- | 18.0 (12.1) | -- |
| Moderate | 14.1 (7.82) | -- | 34.4 (17.6) | -- | 11.8 (8.83) | -- | 29.5 (18.0) | -- |
| Marked | 17.7 (16.2) | -- | 46.1 (22.2) | -- | 20.2 (16.2) | -- | 45.3 (20.5) | -- |
| BI-RADS Breast Density |  | 0.80 |  | 0.79 |  | 0.32 |  | 0.13 |
| BI-RADS A (almost entirely fatty) | 8.03 (9.04) | -- | 20 (15) | -- | 4.30 (4.27) | -- | 13 (9) | -- |
| BI-RADS B | 9.15 (11.1) | -- | 25 (19) | -- | 7.75 (8.80) | -- | 22 (16) | -- |
| BI-RADS C | 9.41 (9.38) | -- | 26 (18) | -- | 7.58 (8.63) | -- | 21 (16) | -- |
| BI-RADS D (extremely dense) | 11.3 (8.59) | -- | 27 (21) | -- | 8.51 (10.5) | -- | 23 (18) | -- |
| P value signify F-test | | | | | | | | |

Supplemental Table 2: Linear regression models of race/ethnicity and log-transformed quantitative BPE

|  | Log-transformed Median BPE | | Log-transformed BPE Ratio | |
| --- | --- | --- | --- | --- |
|  | Unstandardized Β₁, (95% CI) [p-value] | Standardized Β₁, (95% CI) [p-value] | Unstandardized Β₁, (95% CI) [p-value] | Standardized Β₁, (95% CI) [p-value] |
| **Adjusted** |  |  |  |  |
| **Race/ethnicity** |  |  |  |  |
| Black women | 0.22 (0.07, 0.38) [0.003]*** | 0.23 (0.08, 0.39) [0.003]** | 0.077 (-0.052, 0.206) [0.24] | 0.09 (-0.06, 0.24) [0.24] |
| White women | REFERENCE | REFERENCE | REFERENCE | -- |
| **BMI (per 1 kg/m2)** | 2.52e-5 (-4.05e-4, 4.56e-4) [0.91] | 3.31e-03 (-0.05, 0.06)[0.90] | 2.48e-4 (-1.34e-4, 6.31e-4) [0.20] | 0.04 (-0.02, 0.09) [0.20] |
| **Age (per 1 year)** | -0.02 (-0.03, -0.01) [<0.001]*** | -0.26 (0.36, -0.16) [<0.01]* | -0.016 (-0.024, -0.009) [<0.001]*** | -0.22 (-0.31, -0.12) [<0.001]*** |
| **Menopausal Status** |  |  |  |  |
| Premenopausal | REFERENCE | REFERENCE | REFERENCE | REFERENCE |
| Postmenopausal | -0.17 (-0.38, 0.03) [0.10] | -0.18 (-0.39, 0.04) [0.10] | -0.20 (-0.38, -0.02) [0.03]* | -0.23 (-0.44, -0.03) [0.03] |
| **FGT volume (per 1 cm3)** | 7.33e-5 (-3.37e-4, 4.83e-4) [0.73] | 0.01 (-0.05, 0.07) [0.72] | -2.39e-4 (-5.97e-4, 1.19e-4) [0.19] | -0.04 (-0.10, 0.02) [0.19] |
| adjusted for age, BMI, menopausal status, and FGT volume  * denotes statistical significance | | | | |

Supplemental Figure 1. Distribution of BPE Ratio


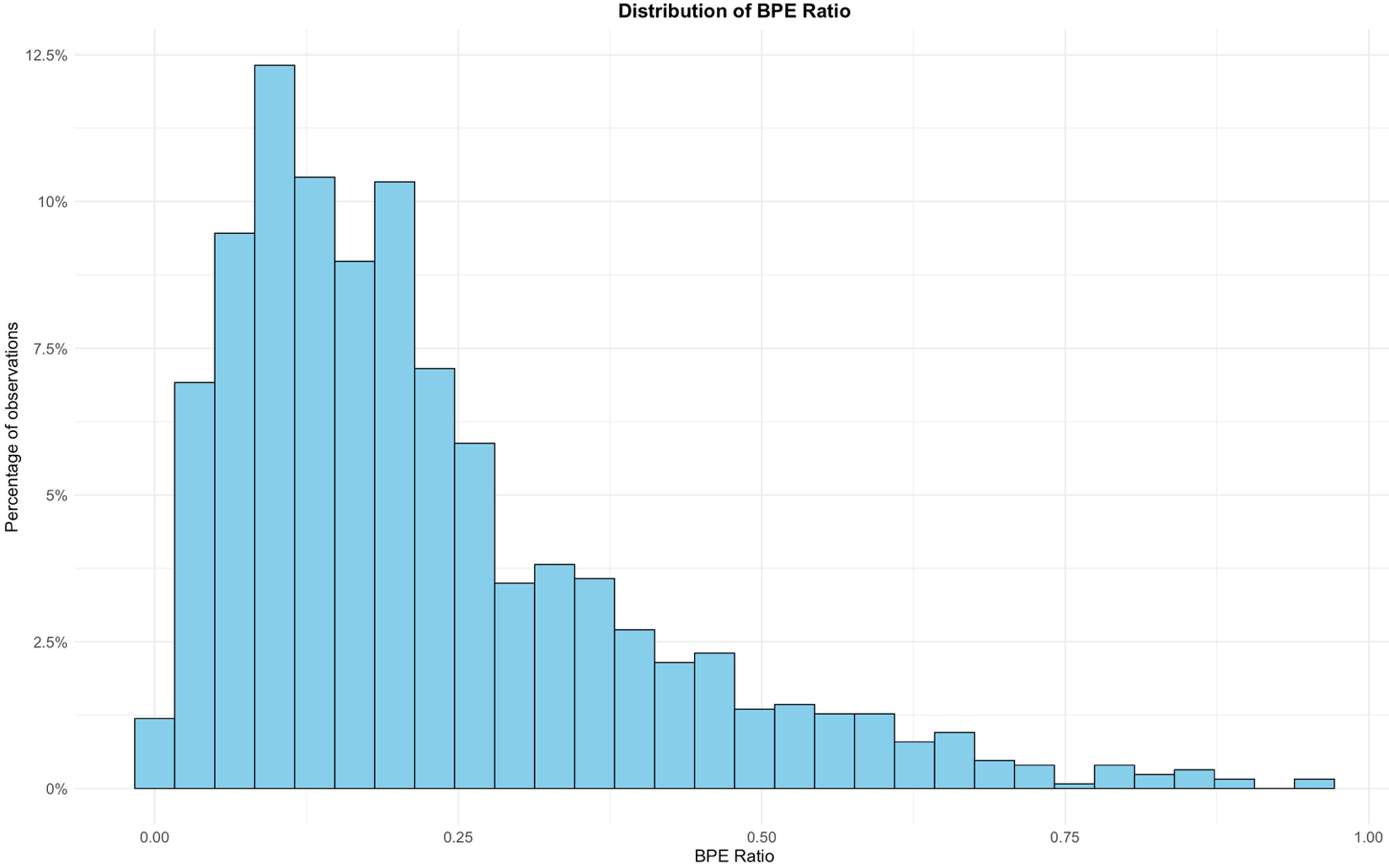


Supplemental Figure 2. Distribution of Median BPE


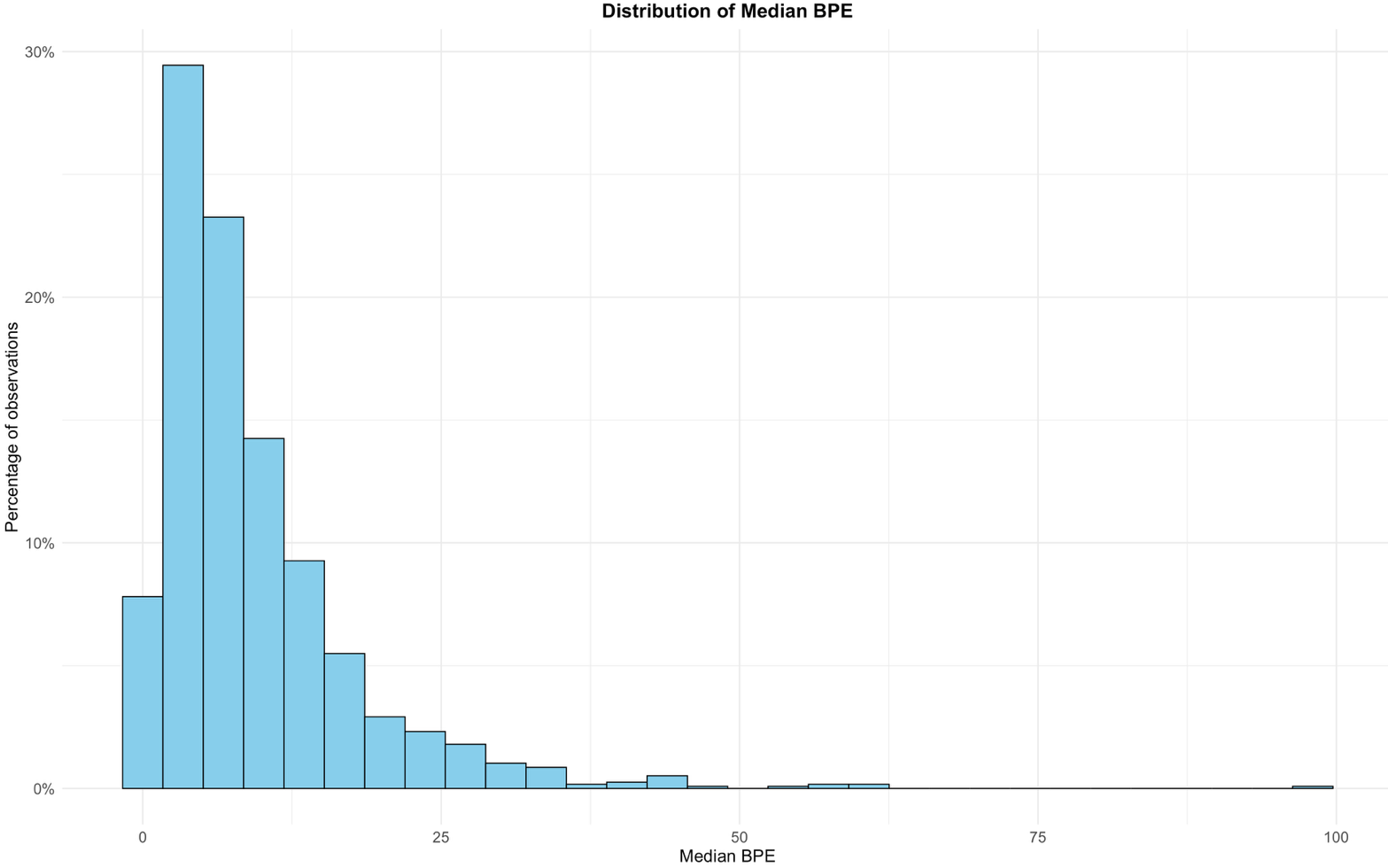


Supplemental Figure 3. Distribution of Log-transformed BPE Ratio


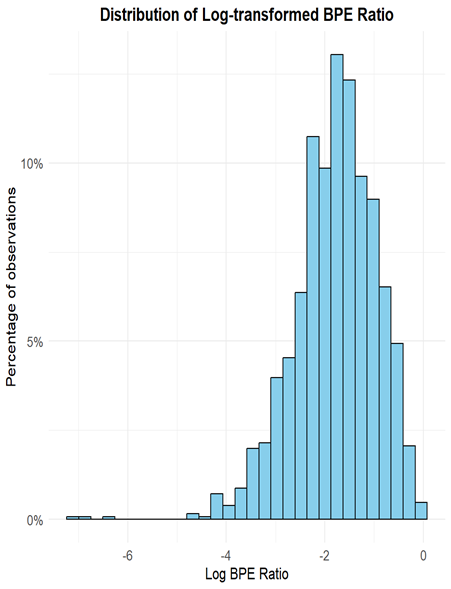


Supplemental Figure 4. Distribution of Log-transformed Median BPE


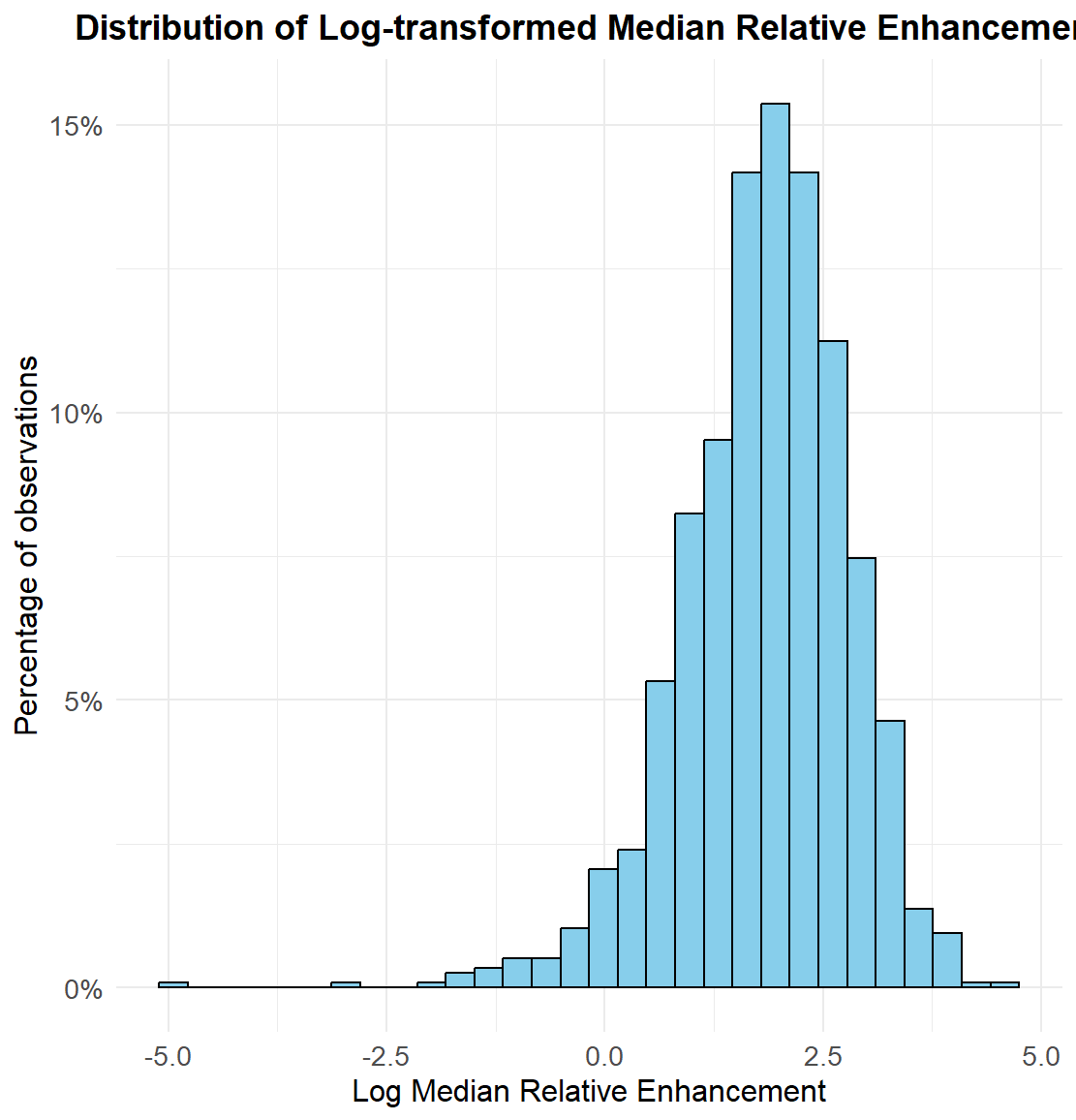

Supplement: Supplementary file 1 — Supplementary Material [file CNCR-131-e70174-s001.docx]
